# Ascending Aortic Pseudostenosis following the Classic Bentall Inclusion Technique

**DOI:** 10.1055/s-0042-1757871

**Published:** 2023-02-27

**Authors:** Andrea Stadlbauer, Jing Li, Natascha Platz Batista da Silva, Christian Stadlbauer, Christof Schmid, Bernhard Floerchinger

**Affiliations:** 1 Department of Cardiothoracic Surgery, University Medical Center, Regensburg, Bavaria, Germany; 2 Department of Radiology, University Medical Center, Regensburg, Bavaria, Germany

**Keywords:** graft inclusion, pseudostenosis, aorta

## Abstract

We present the case of a 52-year-old with a history of aortic valve replacement and replacement of the ascending aorta with the graft inclusion technique presenting with dizziness and collapse. Computed tomography and coronary angiography revealed pseudoaneurysm formation at the anastomotic site causing aortic pseudostenosis. Due to severe calcification of the graft inclusion surrounding the ascending aorta, we performed a redo ascending aortic replacement using a two-circuit cardiopulmonary bypass to avoid deep hypothermic cardiac arrest.

## Introduction

The graft inclusion technique is a surgical approach in patients with aortic aneurysm or dissection which was first described by Bentall and De Bono in 1968. 1 Instead of resecting the diseased aortic tissue, it is wrapped around the new prosthesis to achieve better hemostasis. Since this method was prone to pseudoaneurysm formation, it was soon abandoned and—after development of new surgical materials and techniques, including tissue glue—the open graft implantation technique with complete excision of the diseased aorta was adopted. 2 We report the case of a 52- year-old man with a history of aortic valve replacement (AVR) and replacement of the ascending aorta by the graft inclusion technique, suffering from pseudoaneurysm formation causing ascending aortic stenosis (pseudostenosis).

## Case Presentation

A 52-year-old man was brought to the emergency room with dizziness and confusion after collapse. The patient's medical history included two previous cardiac surgical procedures. At the age of 37 years, he underwent mechanical AVR and supracoronary ascending aortic replacement due to congenital aortic valve insufficiency and a 60-mm aneurysm of the ascending aorta. Four years later, the patient underwent an exchange of the prosthetic valve and aortic prosthesis as well as a patch repair of the noncoronary aortic sinus due to prosthetic valve endocarditis and paravalvular leakage. During both operations, the graft inclusion (Bentall) technique was employed (graft inclusion with side-to-side coronary ostial anastomoses).

**Fig. 1:**
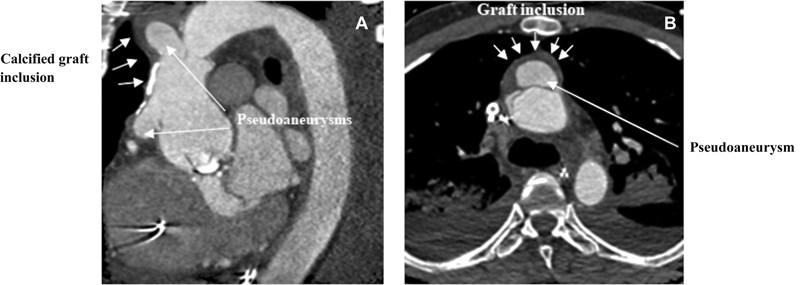
Preoperative computed tomography (CT) scan showing ( **A** ) the calcified graft inclusion and both pseudoaneuysms in parasagittal plane rotated toward the pseudoaneursyms and ( **B** ) the pseudoaneurysm at the distal anastomosis in the transversal plane.

Computed tomography (CT) scan after admission showed no sign of cerebral hemorrhage or ischemia but revealed rupture of the proximal and distal anastomoses of the aortic prosthesis. In the face of ongoing hypoxia refractory to noninvasive ventilation and bilateral pneumonia, the patient was placed on invasive mechanical ventilation before transfer to our institution ( Fig. 1 ).

As the patient required increasing doses of vasopressors and inotropes, venoarterial extracorporeal support (ECLS) was applied via right femoral access. Transthoracic echocardiography (TTE) depicted a new onset of severely reduced left ventricular ejection fraction. The aortic prosthetic valve was competent.

**Fig. 2:**
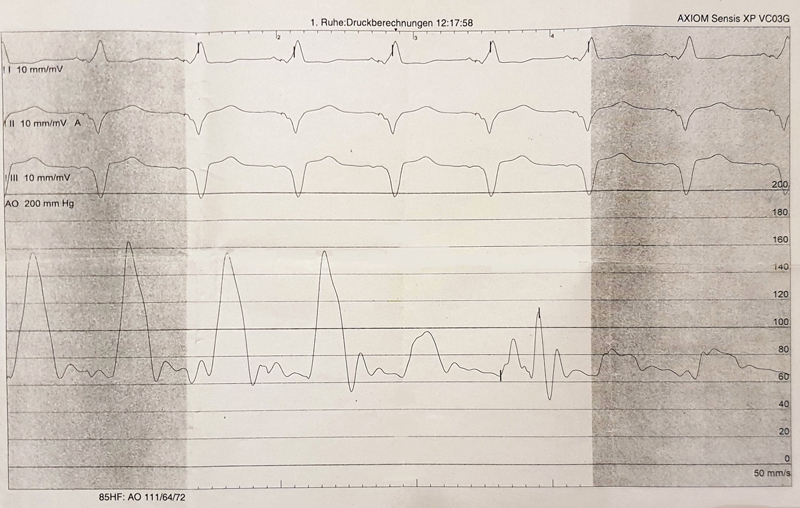
Aortic pressure curve recorded during cardiac catheterization demonstrating the pressure drop at the site of the pseudostenosis.

Cardiac catheterization revealed moderate stenosis of the left anterior descending artery necessitating no intervention. During removal of the angiography catheter, a pressure peak gradient of 60 mm Hg was registered approximately 50 mm above the aortic valve level ( Fig. 2 ). Synopsis of hemodynamic and imaging data implicated a pseudostenosis of the ascending aorta caused by periprosthetic perfusion via proximal and distal leakages into the perigraft space. In the face of these findings and progressive hemodynamic deterioration, we scheduled the patient for surgical revision of the ascending aorta.

**Fig. 3:**
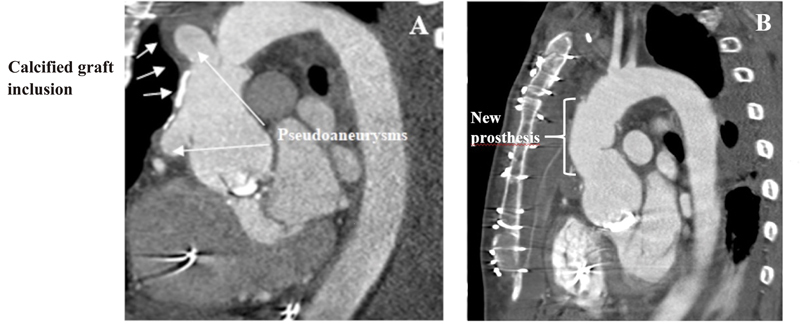
Preoperative computed tomography (CT) scan with ( **A** ) pseudoaneurysms and calcified graft inclusion compared with ( **B** ) postoperative CT scan showing no calcifications.

Under general anesthesia, an arterial cannula was connected to the right subclavian artery via an 8-mm Dacron prosthesis, followed by median sternotomy. Since there was no safe option for aortic cross-clamping due to severe calcifications of the aorta, the aortic arch and brachiocephalic artery were exposed. Cardiopulmonary bypass was carried out using the ECLS cannulas in the right femoral vein and artery with an additional cannula and tubing inserted through the right subclavian artery, creating two arterial blood circuits: oxygenated blood was administered via right subclavian artery for antegrade cerebral perfusion and via right femoral artery for systemic perfusion. A retrograde cardioplegia catheter was inserted into the coronary sinus, followed by separate cross-clamping of the brachiocephalic trunk and the aortic arch distal to the brachiocephalic trunk. Thus, continuous cerebral and systemic perfusion could be maintained during surgery. The patient was cooled to a tympanic temperature of 28°C. After induction of cardioplegic arrest, the severely calcified graft inclusion was excised, revealing the periprosthetic space filled with fresh blood clots. The ascending prosthesis was excised, and a supracoronary aortic replacement was performed using a 34-mm tube prosthesis. The suture lines were both reinforced with Teflon felt. After termination of cardiopulmonary bypass and removal of the subclavian cannula, femoral venoarterial support was maintained. Due to severe coagulopathy, the chest remained open and was closed 2 days later. The patient could be weaned successfully from extracorporeal membrane oxygenation 3 days after surgery. Postoperative TTE showed normal left ventricular ejection fraction and competent aortic valve prosthesis. After prolonged ventilation due to pneumonia, the patient was weaned from the respirator on postoperative day 10. A postoperative CT scan showed the ascending aorta well reconstructed without any signs of prosthetic leakage or new pseudoaneurysm formation. The patient was discharged in good condition 21 days after surgery ( Fig. 3 ).

## Discussion

Pseudoaneurysm formation at the site of an aortic anastomosis is a well-known complication after vascular graft implantation. This may also occur following graft inclusion after aortic replacement. 2 Accumulation of blood in the perigraft space is thought to expose anastomotic suture lines to tension, which may eventuate into anastomotic leakage. Previous studies have shown benefits of the full resection technique, as graft inclusion is a significant independent predictor of early and also late mortality, with higher reoperation rates. 3 4

In our case, the diseased aorta used for wrapping the prosthesis was severely calcified. The expanding pseudoaneurysms at the proximal and distal anastomoses resulted in compressive stenosis of the aortic prosthesis, as demonstrated during angiography, where a pressure drop of 100 mm Hg was measured at the site of distal aortic anastomosis. Due to severe calcification of the graft inclusion, the ascending aorta, and the aortic arch, we used both femoral and subclavian access for arterial inflow, with two circuits, allowing us to clamp the noncalcified aortic arch and to induce cardioplegic arrest with a low risk for cerebral embolization. This technique has already been described and performed for aortic hemiarch replacement by Kim et al. 5 Though several organs (liver, kidney, or spinal cord) are known to have a higher tolerance for ischemia compared with brain tissue, it is well-known, that circulatory arrest is associated with an increased risk for postoperative hepatic and renal failure compared with the risk in patients with continuous perfusion. 6 7 Avoiding circulatory arrest could, therefore, improve the patient's recovery by reducing complications like coagulopathy, acute kidney injury, or neurologic events.

This case demonstrates that graft inclusion after ascending aortic replacement may lead to a pseudostenosis of the aorta caused by anastomotic leakages. If the ascending aorta is not eligible for cross-clamping, avoidance of deep hypothermic cardiac arrest may be achieved by cross-clamping of the proximal aortic arch with additional cannulation of the right subclavian artery for selective cerebral perfusion.
